# Effects of Oil Types and Fat Concentrations on Production Performance, Egg Quality, and Antioxidant Capacity of Laying Hens

**DOI:** 10.3390/ani12030315

**Published:** 2022-01-27

**Authors:** Zhouyang Gao, Zhongyi Duan, Junnan Zhang, Jiangxia Zheng, Fuwei Li, Guiyun Xu

**Affiliations:** 1 Key Laboratory of Animal Genetics and Breeding of the Ministry of Agriculture, National Engineering Laboratory for Animal Breeding, Department of Animal Genetics and Breeding, College of Animal Science and Technology, China Agricultural University, Beijing 100193, China; gaozhouyangcau@163.com (Z.G.); cauzhangjn@163.com (J.Z.); jxzheng@cau.edu.cn (J.Z.); 2 National Animal Husbandry Service, Ministry of Agriculture and Rural Affairs, Beijing 100125, China; dzy806@163.com; 3 Poultry Institute, Shandong Academy of Agricultural Sciences, Jinan 250100, China

**Keywords:** Hyline brown, oils and fats, production performance, nutritional composition, oxidative capacity

## Abstract

**Simple Summary:**

Oils and fats are relevant sources of energy and functional substances in the animal’s body, ensuring its normal growth and development of laying hens needs. Eggs are rich in proteins, amino acids, and fatty acids, and are considered as ‘the ideal nutrient reservoir for humans’. Adding different types and amounts of oils and fats to feed can affect the production performance and egg quality of laying hens. Therefore, it is particularly relevant to investigate the appropriate type and proportion of oils and fats to be used in egg farming. We studied the effects of different concentrations of soybean oil, lard and mixed oils on the production performance, egg quality, and antioxidant substances of laying hens. The results demonstrated that the type and quantities of oils and fats in the diets of laying hens had significant effects on the parameters studied. Thus, this experiment provides a reference for the selection of different types of oils and fats in the egg production process to improve the quality and economic benefits of eggs.

**Abstract:**

In this study, soybean oil, lard and mixed oils were added to the feed in two concentrations (1.5% and 3% of each), resulting in six experimental groups. The control group was fed with a base diet without additions, and used to compare the effects of feeding on production performance and egg quality of laying hens. The results demonstrated that: (1) the 3% supplemented-oils or lard group showed a decrease in laying rate; (2) 1.5% and 3% added-lard significantly increased the total amount of unsaturated fatty acids in eggs, compared to the control group; (3) 1.5% and 3% soybean oil increased the content of mono/polyunsaturated fatty acids, cholesterol, phospholipids and choline in eggs; (4) glutathione peroxidase (GPx) and superoxide dismutase (SOD) contents were increased in all groups, being the most evident in the lard-treated group; (5) all experimental groups showed an increase in the content of essential and non-essential amino acids in albumen; (6) 3% oils, especially the mixed oils, damaged the structure of globules of cooked egg yolks. Therefore, the use of 1.5% soybean oil in the feed diet of Hyline brown hens resulted in the most adequate oil to ensure animal health and economic significant improvements in this experiment.

## 1. Introduction

Oils are the most common source of energy in feed diets for laying hens, affecting energy production, absorption of fat-soluble nutrients, resistance to the heat stress, reduction of dust, and improvement of immunity. Moreover, they improve egg production performance and feed intake [1,2,3,4]. Feeding oils are classified into animal, vegetable, and mixed oils. Animal oils are mainly extracted from subcutaneous adipose tissue [5], and the most common types in production are lard and fish oils. Vegetable oils include soybean, rapeseed, and linseed oils, which are obtained from plant seed kernels after pretreatment, including cleaning, removing impurities, dehulling, crushing and softening, followed by extraction by mechanical pressing or solvent leaching. Afterwards, the resultant crude oil is refined [6,7]. The mixed oil includes different oils according to a defined ratio. The effects of oils on the production performance and egg quality of laying hens have been reported several times [8,9,10,11]. Numerous evidence has shown that under high-temperature conditions, the addition of appropriate amounts of soybean oil to the diet of laying hens, alleviated heat stress and improved egg production and feed conversion ratio, without significant adverse effects on egg weight [12,13]. However, it caused oxidative damage to the body and increased serum malondialdehyde (MDA) levels [14]. Other studies have also shown that adding 4.3% of lard to the diet increased egg production and average weight, with a significant improvement in yolk color [15].

Eggs are an excellent source of animal protein for humans, as they are rich in high-quality protein, fat, cholesterol, and other micronutrients [15]. Several studies have demonstrated that the addition of oils to the diet can affect the nutritional composition of eggs [16,17], including the lipid structure of egg yolk. Thus, the addition of moderate amounts of oils, especially vegetable oil, to diets increase the content of unsaturated fatty acid and reduces the cholesterol content of the yolk [18,19]. However, the addition of 2% cottonseed oil to the diet of laying hens increases the hardness of the yolk, resulting in a so-called “rubber egg”, affecting the taste of it [20].

Although oils have been widely used in poultry, there are several drawbacks to the addition of oils to diets [21,22]. Thus, compared with broilers, the addition of oil in laying hens has to be less, because it may lead to excessive fat deposition, decreased meat quality, unbalanced diet nutrition [23], and feed toxin contamination [24]. As a result, fatty liver hemorrhagic syndrome and metabolic disorders leading to decreased performance and increased mortality in laying hens may occur [25,26]. In addition, the rapid oxidation and rancidity of oils loom the safety of livestock and their byproducts, reduce the production performance, alter the antioxidant status of laying hens, and destroy the structure of egg yolk globules [27]. Previous studies have demonstrated the complexity of adding a single type of oil to support the needs of livestock and poultry growth [28,29]. Thus, different proportions and types of oils need to be added to the feed of laying hens. However, the difficulty to control the effect of mixed oils relies on the mutual influence of different types of oils, their mechanism of action not being fully understood. Therefore, the rational and accurate addition of oils to the feed diet is highly relevant, with a comprehensive analysis of the effects of fed oils on egg production performance, quality, nutrient composition, and oxidative properties of laying hens. In this study, we investigated the production performance of different concentrations of soybean oil, lard, and mixed oils in egg feeds for Hyline brown hens during the experimental period, and comprehensively and systematically determined the nutritional composition and conventional egg quality in albumen and yolks, oxidation indexes, textural characteristics, and microstructure of cooked egg yolks. The aim was to provide a reference for the selection of different types of fats and oils in egg rations in order to improve egg quality and economic efficiency of egg production.

## 2. Materials and Methods

### 2.1. Experimental Materials and Feeding Management

We conducted the tests at the experimental base of the Poultry Institute of Shandong Academy of Agricultural Sciences. A total of 840 Hyline brown laying hens (40 w of age; 2005 ± 130 g) were housed in cages at 23 ± 2 °C. The pre-feeding period and the experimental period lasted for one and four weeks, respectively. The egg production, feed intake, and general health of the hens was observed during the pre-feeding period. Feed was purchased from Beinongda Technology Co., Ltd. (Jinan, China) and soybean, lard, and mixed oils (soybean, soybean-phospholipid, coconut, rice, and antioxidants) were ordered online. According to NRC feeding standards [30], corn and soybean meal were selected as the main diet components in appropriate proportions to form a corn-soybean meal-based diet. The feeds selected for the experiment were made by a small crushing mixer and were powdered compound feeds with coarser crushed material; the oils and fats used for the experiment were kept in a cool and ventilated place to avoid their oxidation and decay, while the performance of the hens was monitored daily to ensure that they had sufficient water and food everyday and eggs were collected daily. The basic diet formulation for laying hens is shown in Table 1.

### 2.2. Experimental Groups and Sampling Time

The experimental hens were randomly divided into seven groups (with 120 animals each, considering three replicates of 40 chickens). The control group was fed with a corn-soybean meal-based diet, and the experimental groups were fed with the same base diet with either 1.5% or 3% of soybean oil, lard, or mixed oils, respectively. Table 2 shows the nutritional composition of the six experimental groups. A sampling of the experimental eggs was carried out on the following day after the end of the experimental period.

### 2.3. Production Performance and Egg Quality Determination

The number of eggs laid, egg weight (EW), and mortality were recorded daily, while feed conversion ratio (FCR), laying rate, average daily feed intake (ADFI), and average EW (AEW) were calculated weekly.

About forty eggs from each group were randomly selected for egg quality determination, including EW, yolk weight (YW), albumen height (AH), yolk color (YC), and Haugh units (HU). These indicators were measured using the EMT-5200 multifunctional egg tester (Robotmation, Tokyo, Japan). The egg shape index (ESI) was calculated with the formula: ESI = egg length/egg width [31]. Eggshell strength (ESS) was measured by an eggshell strength tester Model-II (Robotmation, Tokyo, Japan), and the eggshell weight (ESW) was obtained with an electronic balance (YP601N, Qinghai Co., Ltd., Shanghai, China) after removing the eggshell membrane. Eggshell thickness (EST) was determined in three zones (at both ends and the equator), calculating the average of the three measurements (Robotmation Co., Ltd., Kyoto, Japan). The yolk ratio was calculated as yolk weight/egg weight × 100% [32].

### 2.4. Determination of Nutritional Indicators of Eggs

Determination of the nutritional composition of raw egg yolk: nine eggs were selected from each group (three eggs from each replicate). The raw egg yolk was separated and mixed. The nutrient content and moisture (GB 5009.3-2016) of raw egg yolks were determined following the Chinese food safety standards, including fatty acids (GB 5009.168-2016); proteins (GB 5009.5-2016); cholesterol (GB 5009.128-2016); amino acids (GB 5009.124-2016); and using a kit from Qingdao Sci-Tech Quality Co. (Qingdao, China) for phospholipids (PL). Briefly, this kit applies the double antibody sandwich method. A solid antibody phase was made with a purified PL antibody. Samples were added to the microtiter wells coated with the monoclonal antibody and combined with the HRP-labeled PL antibody to form an antibody–antigen–enzyme-labeled antibody complex. The color was developed by adding the tetramethylbenzidine (TMB) substrate and converted to blue and to the final yellow. The color shade is positively correlated with the PL content in the sample. The absorbance was measured at 450 nm using an enzyme standardization instrument. The PL concentration in egg yolk was calculated based on the standard curve. Data from three parallel-samples were obtained for each experimental group.

Determination of the nutritional composition of albumen: nine eggs were selected from each group (three eggs from each replicate). Albumen was mixed, and the nutrient content and moisture were determined according to the Chinese food safety standard, following the guidelines named above. Data from three parallel samples were obtained for each experimental group.

### 2.5. Determination of Quality Indicators in Cooked Egg Yolk

The textural analysis involved the random selection of 15 to 20 eggs from each experimental group. The freshly collected eggs were refrigerated at −20 °C for one week and pretreated a week later. The pretreatment procedure was as follows: all eggs were boiled for 10 min and cooled to room temperature to prepare cooked yolks. The yolks were manually separated from the shells and albumen and the intact yolks were placed on a test plate for a double compression test. The analysis of the textural parameters of the cooked egg yolks included hardness, springiness, cohesiveness, gumminess, chewiness, and resilience. The SMS P50 probe was used for the texture analyzer with the following parameters set: pre-squeeze rate 0.50 mm/s, test rate 0.50 mm/s, post-test rate 0.50 mm/s, interval time 5 s, and trigger force 5.0 g.

### 2.6. Sample Preparation for Scanning Electron Microscopy (SEM) Observations

Three eggs were randomly selected from each experimental group and were prepared for microstructure observation according to the method above. The process was as follows: (1) Sample preparation and fixation: the cooked egg yolk was cut into a central slice of 6 mm × 6 mm × 4 mm. The samples were fixed in 2.5% glutaraldehyde (pH 7.2–7.4) for 4–6 h to prevent from disintegration during the experiment. (2) Dehydration: to remove the fixative, samples were washed four times with PBS (25 min each). Samples were dehydrated with different concentrations of ethanol solution in a stepwise manner (20 min each wash), (i) two washes of 30% (*v*/*v*), (ii) two washes of 50% (*v*/*v*), (iii) one wash of 70% (*v*/*v*), (iv) one wash of 90% (*v*/*v*), and (v) two washes of 100% (*v*/*v*). (3) Drying: the dehydrated cooked egg yolk samples were immersed in isoamyl acetate for 20 min by three times, and transferred to beakers for drying [33]. (4) Spraying: the dried samples placed on a sample table were sprayed with gold using an IB-3 ion-sputtering instrument [34]. (5) On-board observation: the prepared samples were placed into a JEM-840 SEM (JEOL Corporation, Osaka, Japan). The samples were observed and photographed at different magnetization rates (200×, 500×, 1000×).

### 2.7. Determination of Raw Egg Yolk Oxidation Index

Determination of yolk oxidation index: nine eggs were selected from each group (three eggs from each replicate), and refrigerated at 4°C for one week. Then, the yolks were separated to determine MDA content, and superoxide dismutase (SOD) and glutathione peroxidase (GPx) activities. MDA content was determined according to the Chinese food safety standard (GB 5009.181-2016); SOD activity was determined with a commercial kit from Qingdao Sci-Tech Quality Testing Co., (Qingdao, China); and GPx was determined using the same method as for PL determination.

### 2.8. Statistical Analysis

In all statistical analyses, each replicate was considered as a test unit. To identify the effects of types and concentrations on production performance and egg properties, data were analyzed using a two-way mixed-design analysis of variance (ANOVA) by using SPSS 20.0 (SPSS, Chicago, IL, USA) [35]. Test data results were expressed as mean ± SD and were considered significantly different at *p* < 0.05. GraphPad Prism 9 (GraphPad Software Inc., San Diego, CA, USA) was used for graph design.

## 3. Results

### 3.1. Performance of Laying Hens

Table 3 shows the results of the performance of laying hens. To test the effects of types and concentrations on production performance, two-way mixed design analysis of variance (ANOVA) revealed a significant effect of types and concentrations on the main and interaction effects of laying rates. The results demonstrated that the laying rate (LR) was significantly lower (*p* < 0.05) in hens fed with 3% lard, compared to those fed with 1.5% lard. The addition of lard and mixed oils caused a decrease in LR compared to the control group (*p* < 0.05). Although the addition of 3% mixed oils caused the greatest decrease in LR (*p* < 0.05), the addition of 1.5% soybean oil showed no significant difference. Moreover, there were no significant differences in FCR, AEW, and ADFI between groups with different content of oils during the experimental period.

### 3.2. Egg Quality

Table 4 shows the results of egg quality measurements. To test the effects of types and concentrations on egg quality, two-way mixed design analysis of variance (ANOVA) revealed a significant effect of types and concentrations main and interaction effects on HU, YC. The results indicated that there were no significant differences in EW, ESI, ESS, yolk percentage (YP), eggshell thickness (EST), and eggshell weight (ESW) between different experimental groups. However, HU and YC were differentially affected (*p* < 0.05). Compared to the control group, the addition of soybean oil and lard at different concentrations improved YC (*p* < 0.05), with 1.5% soybean oil resulting in the highest YC increase (*p* < 0.05). The addition of lard did not significantly differ (*p* < 0.05) in protein content and HU, but soybean oil decreased HU, (*p* < 0.05), compared to the control group.

### 3.3. Measurement of Nutritional Indicators of Eggs

Table 5 shows the results of fatty acids from raw egg yolks. To test the effects of types and concentrations on the nutrition of raw egg yolk, a two-way mixed design analysis of variance (ANOVA) revealed a significant effect of types on C14:0 and C20:3n6. A significant effect of concentrations on C16:0, C17:0, C18:0, C18:1n9c, C18:3n6, C20:2, and C22:0 and a significant effect of types and concentrations on the main and interaction effects of C15:0, C16:1, C18:2n6c, C18:3n3, C20:1, and UFA.

Fatty acids content: in raw egg yolk, the saturated fatty acids with the higher contents were C16:0 (palmitic acid) and C18:0 (stearic acid); while the unsaturated fatty acids with the higher contents were C16:1 (palm oleic acid), C18:1n9c (oleic acid), C18:2n6c (linoleic acid), and C20:4n6 (arachidonic acid). Moreover, the saturated fatty acid C14:0 (myristic acid), and the unsaturated fatty acids C16:1, C18:1n9c, C18:2n6c, C18:3n3 (α-linolenic acid), C20:1 (eicosanoic acid), and C20:2 (eicosadienoic acid) were remarkably affected by the addition of oils (*p* < 0.05).

Compared with the control group, the lard addition significantly increased the content of C18:1n9c, C14:0, C17:0 (margaric acid), C18:3n3, and C20:1 (*p* < 0.05), while soybean (3%) and mixed oils increased the content of C18:2n6c, C18:3n3, C20:2, and C20:3n6 (methyl linoleate) in yolk (*p* < 0.05). In summary, while there were no significant differences in the total saturated fatty acid content of egg yolk among all the experimental groups, the addition of lard had significantly increased the content of unsaturated fatty acids in egg yolk.

Table 6 shows the results of amino acids, proteins, moisture, cholesterol, choline, phospholipids from raw egg yolks. The two-way mixed design analysis of variance (ANOVA) revealed a significant effect of types on Cys and phospholipid, as well as a significant effect of concentrations on Thr, Glu, Val, Ile, Tyr, Lys, His, Arg, and cholesterol and a significant effect of types and concentrations of the main and interaction effects of Asp, Ser, Gly, Ala, Met, and choline. Amino acids content in egg yolk: the main amino acids present in raw egg yolk were aspartic acid (Asp), glutamic acid (Glu), leucine (Leu), and methionine (Met), the latter of which was the most variable one. The addition of mixed oils increased the content of cysteine (Cys) compared to the control group (*p* < 0.05). Meanwhile, 1.5% of mixed oils significantly increased the content of essential amino acids (*p* < 0.05); the addition of soybean oil significantly increased the content of cholesterol and choline in egg yolk (*p* < 0.05). The content of PL in egg yolk was increased by the addition of oils in all groups, resulting in the highest content of PL after the addition of mixed oils (*p* < 0.05). The content of Met was significantly decreased in all experimental groups (*p* < 0.05).

The two-way mixed design analysis of variance (ANOVA) revealed a significant effect of types on Gly, Ala, Ile, Arg, and moisture and a significant effect of types and concentrations on the main and interaction effects of Asp, Thr, Ser, Glu, Cys, Val, Met, Leu, Tyr, Phe, Lys, and Pro. Amino acid content in albumen: Table 7 shows that the most abundant amino acids in raw albumen were Asp, Glu, and Leu. Different diets had distinct effects on the content of amino acids, proteins, water, cholesterol, choline, and PL in raw albumen. We found that the variation in types and amounts of oil had a significant effect, mainly on Thr, Ser, Glu, Val, Ile, and Leu (*p* < 0.05).

Essential and non-essential amino acids were significantly increased (*p* < 0.05) in each experimental group compared to the control. Mainly, 1.5% and 3% lard experimental groups showed the highest increase in the content of essential and non-essential amino acids, which were determined in albumen (*p* < 0.05).

### 3.4. Texture Profile Analysis of Cooked Egg Yolk

Table 8 displays the textural parameters of cooked egg yolks. Two-way mixed design analysis of variance (ANOVA) revealed a significant effect of type concentrations on hardness, gumminess, chewiness, and resilience. The addition of 3% soybean oil, 3% lard, and 1.5% mixed oils significantly increased the hardness of cooked egg yolks compared to the control group (*p* < 0.05). However, the addition of 1.5% soybean oil did not affect the hardness of cooked egg yolks compared to the controls. Compared with the control group, adding different types and concentrations of oils significantly reduced the egg’s resilience. Moreover, 3% soybean oil reduced springiness, and 1.5% of soybean oil significantly increased gumminess.

### 3.5. Microstructure of Cooked Yolk

**Figure 1:**
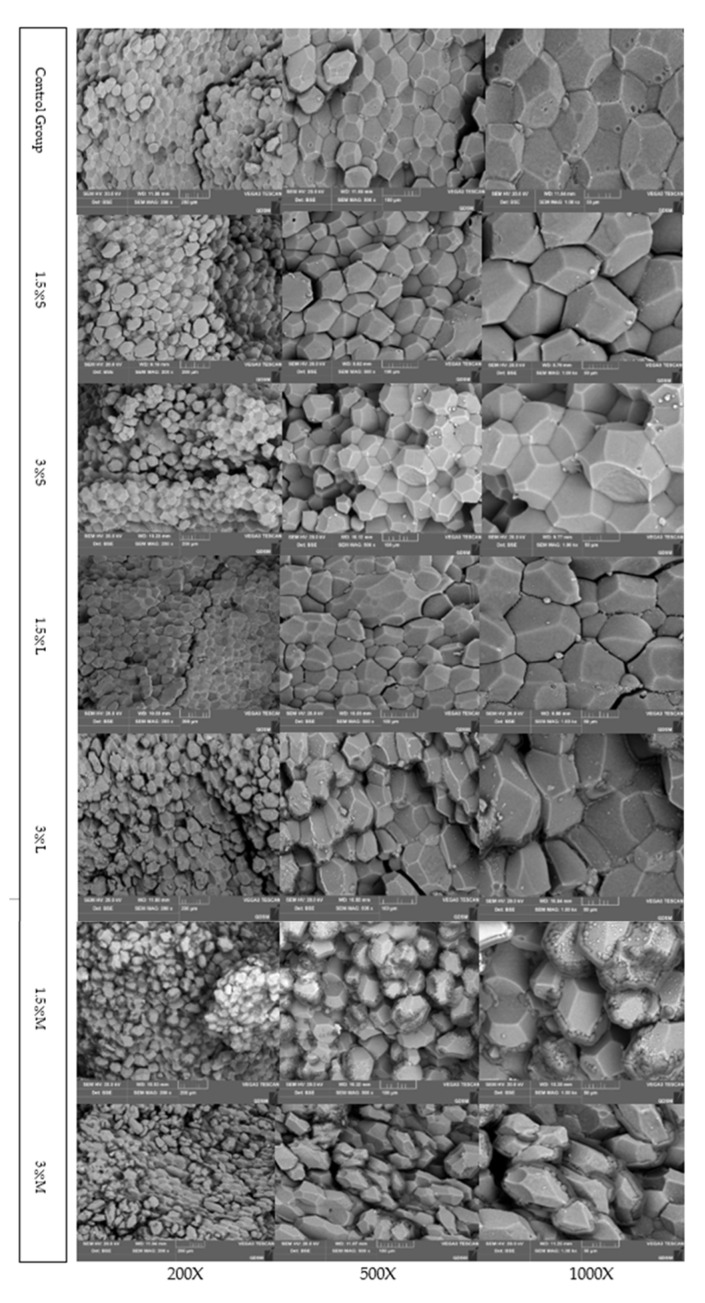
Micrographs of cooked yolk observed by JEM-840 at different magnifications. The abscissa indicates the different magnifications. A 1.5% S or 3% addition of 1.5% or 3% of soybean oil to the base diet, respectively. A 1.5% L or 3% addition of 1.5% or 3% of lard to the base diet, respectively. A 1.5% or 3% M: addition of 1.5% or 3% of mixed oils to the base diet, respectively.

The microstructure of cooked yolk was observed by SEM, to study the effect of the oils added to the feed on the quality of eggs at the sub-microscopic level. The cooked yolk is composed of polyhedral-shaped yolk balls with a loose texture and a continuous undulating appearance. Under normal conditions, different regions of the yolk spheres were combined without gaps or cross-linking, and the yolk spheres consisted of embedded spheres and holes with uneven and angular surfaces and edges. Figure 1 shows the SEM results. Although the addition of 1.5% oils did not affect the yolk-sphere structure, 3% oil damaged the structure, compared to the control group. Moreover, the addition of mixed oil significantly damaged the structure of the yolk spheres, with evident signs of cross-linking and fragmentation between the yolk spheres. Furthermore, the damage of the yolk spheres increased with the oil concentration.

### 3.6. Oxidative Stability of Egg Yolk

**Figure 2:**
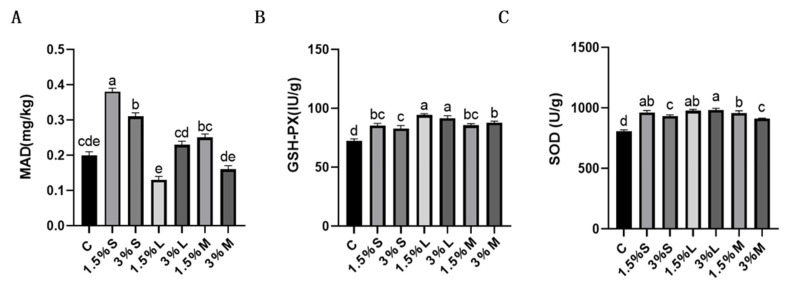
Each value is presented as mean ± SD, different superscript (a–e) indicates significant differences (*p* < 0.05). The effect of different oils on MDA, SOD, and GPX in egg yolk. C: control group; 1.5% S or 3%: addition of 1.5% or 3% of soybean oil to the base diet, respectively; 1.5% L or 3%: addition of 1.5% or 3% of lard to the base diet, respectively; 1.5% or 3% M: addition of 1.5% or 3% of mixed oils to the base diet, respectively. (**A**): effect of different oils and fats on MDA in raw egg yolk; (**B**): effect of different oils and fats on GSH-PX in raw egg yolk; (**C**): effect of different oils and fats on SOD in raw egg yolk.

The levels of oxidative indicators, such as MDA, GPx, and SOD were also studied in egg yolk (Figure 2). Although 1.5% and 3% lard diets significantly increased the content of antioxidant enzymes (*p* < 0.05), the MDA content of the lard and mixed oil groups was similar to that of the control group. However, soybean oil increased the content of MDA (*p* < 0.05).

## 4. Discussion

In this study, we found that after four weeks of the consumption of oil-feeding diets, the laying rate of laying hens was affected. At present, it is essential to add 1% of oil to the basic diet of laying hens, to produce energy-rich formulations and nutrient-rich eggs [36]. The effect of oils on the performance of laying hens is influenced by the type of oils, the amount added, and the nutritional level [37,38]. Hence, the addition of different types and quantities of oils to feed have various effects on the production performance and egg quality of laying hens. The addition of oils in high amounts can induce alterations, such as diarrhea, in livestock and poultry; and high quantities of cottonseed oil can significantly reduce the production performance of laying hens and affect their health [39]. Therefore, it is particularly important to add adequate types and amounts of oils to the diet [40].

In the same experimental oil group, the addition of higher levels of oils decreased the laying rate compared to lower levels of oils. In accordance with previous studies, the addition of low amounts of oils had no effect, but a higher oil content considerably affected the laying rate. Moreover, high quantities of fat intake cause metabolic burden with increased fat accumulation in the liver, which leads to a decrease in laying rate [41,42]. Noteworthy, the addition of different types and quantities of vegetable oils reduced the laying rate, but had no effect on FCR, AEW, or ADFI [12]. Additionally, animal oils decreased the laying rate of hens to different extents, as observed previously [12,43]. This may be because vegetable oils or nutrient sources rich in polyunsaturated fatty acids (PUFA) reduce liver fat content or mitigate the effect on fatty liver syndrome in laying hens, whereas animal oils increase liver lipid deposition and aggravate fatty liver syndrome in laying hens, ultimately leading to a decrease in egg production [44,45].

The color of egg yolk depends on the pigment content of the feed for the laying hens [46]. This pigment, which is fat-soluble, is absorbed and transported to the yolk with fat. Thus, the addition of appropriate amounts of oils aids the absorption and transport of pigments [47]. As in previous studies, we found that the addition of different amounts of soybean oil and lard to the diet significantly improved yolk color compared to the control group [15,48]. However, the mixed oils had no effect on yolk color. The differences in EW, ESS, albumen height, YP, EST, and ESW were similar among different oil groups. Noteworthy, the reduction of egg Haugh units in 1.5% and 3% soybean oil groups needs further investigation [49].

Lipids play an essential role in animal growth and are one of the main components of egg yolk in laying hens [50,51]. Lipid metabolism in the body of laying hens is high after the laying starts, in the peak laying period. Forage lipids are introduced into eggs through the ovaries [52]. Thus, the composition and proportion of FA in forage lipids affect the composition and proportion of FA in egg yolk [53]. Fatty acids can be divided into saturated, monounsaturated, and PUFA, the contents of which are relevant to evaluating the nutritional value of poultry eggs [54]. PUFA are divided into n-3 and n-6 PUFA, with n-3 PUFA being the most beneficial to humans. These benefits include maintaining the relative fluidity of cell membranes [55], esterifying cholesterol, reducing cholesterol and triglycerides levels in serum and liver [56], preventing cardiovascular diseases [57], and modulating immunity and gene expression [58]. We found that the addition of different amounts of soybean oil significantly increased the content of certain n-6 PUFA (C18:2n6c, C18:3n6, and C20:3n6) and n-3 PUFA (C18:3n3), and decreased the total content of saturated fatty acid in egg yolk, compared to the control group. The different amounts of lard increased the total unsaturated fatty acid content in egg yolk compared to the other experimental groups. These findings are consistent with other studies [59,60,61]. Moreover, the addition of soybean oil significantly increased the cholesterol content, choline, and PL in egg yolk compared to the other experimental groups. Although cholesterol might be associated with the risk of coronary heart disease (CHD), only one-third of cholesterol is ingested with food. Thus, the consumption of two eggs a day does not increase cholesterol levels, drawing back the conception of eggs and cholesterol content [49].

Eggs contain high-quality proteins and are a source of amino acids, mainly essential ones, which are rapidly digested and absorbed by the body [62]. Amino acids are divided into essential and non-essential ones, the former of which cannot be synthesized and must be obtained with the diet [63]. Non-essential amino acids are synthesized in animals’ bodies [64]. Previous research has been focused on studying the effect of oil additions on the nutrient content of egg yolks [65]. Thus, we studied the content of the main nutrients, including essential and non-essential amino acids, proteins, and water in albumen. The results demonstrated that the addition of different quantities of soybean oil, lard, and mixed oils to the diet increased the total content of both essential and non-essential amino acids in albumen. Further investigations are needed, as the effect of lard was highly evident.

Textural polyhedral analysis (TPA) assay was conducted in equipment that simulates the chewing of food by human teeth and responds to the textural structure of food perceived by the teeth and palate in the mouth according to the pressure detected by the probe [66]. In this study, the TPA test on cooked egg yolks included the following indicators: hardness, springiness, cohesiveness, gumminess, chewiness, and resilience. The results demonstrated that the hardness of cooked egg yolks increased in all experimental groups, but was similar between the 1.5% soybean oil and the control groups. The texture of eggs play a key role in egg processing and pastry making. The greater the value of springiness and resilience, the better the food quality, the refreshing taste, and the non-stickiness, which is positively correlated with the quality of the cake. However, there was a negative correlation between the value of hardness, chewiness, and the lack of springiness with the quality of the cake [67]. Considering the above, 1.5% soybean oil does not affect the characteristics of eggs. Through scanning electron microscopy, we can find that the yolk was composed of spheres, where the main components were low-density lipoprotein (LDL), high-density lipoprotein (HDL), and yolk globular protein [66,68]. The round balls observed on the surface of the yolk spheres have a fibrous network structure, assumed from the shape and size of these balls, resembling grains. The network structure is composed of LDL and yolk globulin. From the results, it is clear that the addition of high concentrations of oils exacerbates the structural damage of cooked egg yolks.

MDA, the end-product of lipid oxidation, is involved in lipid peroxidation [69]. During intensive breeding, lipid oxidation of feed is a common factor generating oxidative stress in animals [12], having a negative effect on the production performance and egg quality of laying hens. However, the organisms have developed systems to cope with oxidative stress, mainly, a group of enzymes, including SOD and GPx. Many studies demonstrated that the addition of different types of oils to feeds improves the antioxidant capacity of laying hens [70,71]. However, the antioxidant properties of oils on eggs need further studies.

Mixed oils are different oils and fats that are mixed in a certain proportion, and are also a new research hotspot in the field of feed oils and fats in recent years [72]. Previous studies have demonstrated that adding a single type of oil or fat to feed is difficult to meet the needs of livestock and poultry for a variety of FAs, especially EFAs. Therefore, some scholars began to study the mixing of different ingredients in a certain proportion, so that different components have complementary effects, and developed new products [58,73,74]. However, due to the interaction of different types of ingredients in mixed oils, its effect becomes difficult to control, and because the study of the relevant mechanism of action is in-depth enough, it is difficult to determine and explain its effect. This study demonstrated that mixed oils produced an evident damage to the structure of egg yolk and reduced the egg production. While the addition of 1.5% soybean oil to the diet increased the content of nutrients and antioxidant enzymes in eggs, the addition of lard caused a reduction in egg production rate and fatty liver formation in hens. In this study, the effects of three different types and concentrations of soybean oil, lard, and mixed oils were compared to provide scientific relevant data for production practices and dietary health guidelines for laying hens.

## 5. Conclusions

In summary, as the composition of different types of oils is variable, the effects on the production performance, egg quality, and antioxidant capacity in laying hens are diverse and there was also a significant synergistic effect of the concentration and type of oil. Therefore, the choice of oils in the diet feed should be based on studies ascribing the effects and needs of oils in egg production in poultry. Therefore, we recommend adding 1.5% soybean oil to feed diets.

## Figures and Tables

**Table 1:** Ingredients and composition of the basal diet.

Items	%
Ingredient	
Corn	61
Soybean meal	24
Wheat bran	2.5
Stone powder	8.5
Premix ^1^	4
Nutrient composition	
AME (MJ/Kg)	12.47
Crude protein (g/100 g)	16
Methionine (g/100 g)	0.2
Lysine (g/100 g)	0.75
Calcium (mg/kg)	3.16 × 10^4^
Phosphorus (mg/kg)	3.21 × 10^3^
NaCl (g/100 g)	0.3
Moisture	≤10

^1^ Premix: Vitamin premix provided per kg of diet: vitamin A, 200,000 IU, vitamin D_3,_ 100,000 IU, vitamin E ≥ 300 IU, vitamin K_3_ ≥ 60 mg, vitamin B_1_ ≥ 45 mg, vitamin B_2_ ≥ 154 mg, vitamin B_6_ ≥ 61 mg, Niacin ≥ 700 mg, folic acid ≥ 21.9 mg, pantothenic acid ≥ 241.5 mg. Mineral premix provided per kg of diet: Mn 2.5 g, Zinc 2 g, Fe 15.75 g, Cu 0.4 g, I 20 mg, Se 10 mg.

**Table 2:** Ingredients and composition of the experimental diets (as fed-basis).

Ingredients (g/kg of Diet)	Groups ^1^					
	Soybean Oil (1.5%)	Soybean Oil (3%)	Lard (1.5%)	Lard (3%)	Mixed Oils (1.5%)	Mixed Oils (3%)
NaCl (g/100 g)	0.3	0.27	0.37	0.3	0.27	0.29
AME (MJ/kg)	13.57	13.78	13.40	13.69	13.46	13.47
Crude protein (g/100 g)	17.2	17.1	16.8	17.1	17.2	17.3
Methionine (g/100 g)	0.15	0.18	0.12	0.23	0.2	0.2
Lysine (g/100 g)	0.56	0.47	0.49	0.69	0.74	0.78
Calcium (mg/kg)	4.81 × 10^4^	3.34 × 10^4^	3.56 × 10^4^	3.83 × 10^4^	3.00 × 10^4^	3.09 × 10^4^
Phosphorus (mg/kg)	4.39 × 10^3^	4.84 × 10^3^	4.06 × 10^3^	4.70 × 10^3^	4.41 × 10^3^	5.16 × 10^3^

^1^ Soybean oil 1.5% or 3%; addition of 1.5% or 3% of soybean oil to the base diet, respectively. Lard 1.5% or 3%; addition of 1.5% or 3% of lard to the base diet, respectively. Mixed oils: 1.5% or 3%; addition of 1.5% or 3% of mixed oils to the base diet, respectively.

**Table 3:** Effects of different concentrations of soybean oil, lard, and mixed oils on the production performance of laying hens ^1^.

Traits ^2^	Control Group	Concentration ^3^						*p*-Value from ANOVA Mixed Design		
		1.5%			3%					
		Soybean Oil	Lard	Mixed Oils	Soybean Oil	Lard	Mixed Oils	Type	Concentration	Type × Concentration
LR, %	87.78 ± 2.90 ^a^	89.26 ± 0.89 ^a^	83.54 ± 2.46 ^b^	83.66 ± 2.55 ^b^	84.02 ± 0.62 ^b^	81.32 ± 1.99 ^c^	77.93 ± 2.90 ^d^	0.158	0.038	0.023
FCR	1.86 ± 0.14	1.89 ± 0.08	1.89 ± 0.11	1.88 ± 0.12	1.86 ± 0.12	1.86 ± 0.09	1.84 ± 0.13	0.868	0.254	0.124
AEW, g	61.63 ± 1.98	60.56 ± 1.48	60.73 ± 1.42	61.40 ± 1.85	61.41 ± 1.86	61.35 ± 1.84	61.44 ± 1.87	0.089	0.245	0.287
ADFI, g	115.00 ± 3.77	114.32 ± 2.67	114.36 ± 2.58	115.22 ± 1.08	113.88 ± 1.69	114.06 ± 3.86	113.02 ± 1.99	0.189	0.602	0.300

^1^ Each value is presented as mean ± SD; different superscript (a–d) indicates significant differences (*p* < 0.05). ^2^ LR = laying rate; FCR = feed conversion ratio; AEW = average egg weight. ^3^ Soybean oil 1.5% or 3%: addition of 1.5% or 3% of soybean oil to the base diet, respectively. Lard 1.5% or 3%: addition of 1.5% or 3% of lard to the base diet, respectively. Mixed oils: 1.5% or 3%: addition of 1.5% or 3% of mixed oils to the base diet, respectively.

**Table 4:** Effect of different concentrations of soybean oil, lard, and mixed oils on egg quality of laying hens ^1^.

Traits ^2^	Control Group	Concentration ^3^						*p*-Value from ANOVA Mixed Design		
		1.5%			3%					
		Soybean Oil	Lard	Mixed Oils	Soybean Oil	Lard	Mixed Oils	Type	Concentration	Type × Concentration
EW, g	61.15 ± 2.93	60.93 ± 2.85	61.20 ± 2.84	61.19 ± 2.71	61.05 ± 2.48	60.63 ± 3.47	62.28 ± 2.60	0.320	0.432	0.087
ESI	1.29 ± 0.03	1.30 ± 0.04	1.29 ± 0.04	1.28 ± 0.04	1.29 ± 0.03	1.27 ± 0.03	1.29 ± 0.04	0.125	0.080	0.102
ESS, N/cm ^2^	4.72 ± 0.73	4.57 ± 0.71	4.56 ± 0.87	4.40 ± 0.71	4.74 ± 0.71	4.34 ± 0.73	4.86 ± 0.64	0.218	0.381	0.089
AH, mm	7.27 ± 0.84 ^abc^	6.90 ± 0.63 ^c^	7.59 ± 0.90 ^a^	7.37 ± 0.64 ^ab^	7.20 ± 0.67 ^abc^	7.37 ± 0.85 ^ab^	7.12 ± 0.78 ^bc^	0.075	0.128	0.024
HU	83.70 ± 5.90 ^a^	78.87 ± 7.26 ^c^	84.35 ± 6.65 ^a^	83.14 ± 7.14 ^ab^	80.04 ± 7.94 ^bc^	81.96 ± 8.34 ^abc^	79.59 ± 7.36 ^bc^	0.076	0.013	0.032
YC	5.18 ± 0.78 ^d^	6.05 ± 0.79 ^a^	5.67 ± 0.93 ^abc^	5.45 ± 0.88 ^cd^	5.69 ± 0.88 ^abc^	5.93 ± 0.75 ^ab^	5.55 ± 0.64 ^bcd^	0.034	0.178	0.045
YP, %	25.88 ± 1.89	26.14 ± 1.75	26.18 ± 1.88	26.10 ± 2.00	26.24 ± 1.71	26.76 ± 1.82	26.30 ± 1.76	0.132	0.580	0.658
EST, μm	344.19 ± 19.67	347.38 ± 19.83	339.41 ± 28.24	342.19 ± 21.35	350.57 ± 23.13	340.07 ± 28.65	355.72 ± 20.88	0.236	0.400	0.502
ESW, g	6.75 ± 0.39	6.74 ± 0.47	6.79 ± 0.53	6.51 ± 0.38	6.75 ± 0.39	6.70 ± 0.39	6.84 ± 0.51	0.126	0.286	0.346

^1^ Each value is presented as mean ± SD, different superscript (a–d) indicate significant differences (*p* < 0.05). ^2^ EW = egg weight, ESI = eggshell index, ESS = eggshell strength, AH = albumen height, HU = haugh unit, YC = yolk color, YP = yolk percentage, EST = eggshell thickness, and ESW = eggshell weight. ^3^ Soybean oil 1.5% or 3%: addition of 1.5% or 3% of soybean oil to the base diet, respectively. Lard 1.5% or 3%: addition of 1.5% or 3% of lard to the base diet, respectively. Mixed oils: 1.5% or 3%: addition of 1.5% or 3% of mixed oils to the base diet, respectively.

**Table 5:** Effects of different concentrations of soybean oil, lard, and mixed oils on fatty acids of raw egg yolk ^1^.

Traits ^2^	Control Group	Concentration ^3^						*p*-Value from ANOVA Mixed Design		
		1.5%			3%					
		Soybean Oil	Lard	Mixed Oils	Soybean Oil	Lard	Mixed Oils	Type	Concentration	Type × Concentration
C14:0, mg/g	0.81 ± 0.007 ^c^	0.70 ± 0.019 ^e^	0.84 ± 0.004 ^b^	0.76 ± 0.003 ^d^	0.71 ± 0.002 ^e^	0.87 ± 0.019 ^a^	0.78 ± 0.022 ^d^	0.000	0.016	0.639
C14:1, mg/g	0.24 ± 0.032 ^a^	0.12 ± 0.010 ^c^	0.20 ± 0.026 ^ab^	0.20 ± 0.031 ^ab^	0.12 ± 0.009 ^c^	0.20 ± 0.043 ^ab^	0.19 ± 0.015 ^b^	0.000	0.862	0.883
C15:0, mg/g	0.11 ± 0.007 ^cd^	0.09 ± 0.008 ^e^	0.13 ± 0.001 ^a^	0.12 ± 0.005 ^ab^	0.11 ± 0.002 ^bc^	0.10 ± 0.003 ^de^	0.11 ± 0.006 ^cd^	0.005	0.010	0.000
C16:0, mg/g	72.91 ± 0.860 ^a^	67.16 ± 0.831 ^d^	71.26 ± 0.401 ^bc^	70.64 ± 1.023 ^c^	66.87 ± 0.582 ^d^	70.45 ± 0.452 ^c^	72.11 ± 0.863 ^ab^	0.000	0.733	0.048
C16:1, mg/g	10.87 ± 0.108 ^a^	7.27 ± 0.101 ^e^	8.86 ± 0.049 ^c^	8.29 ± 0.164 ^d^	6.67 ± 0.101 ^f^	8.10 ± 0.087 ^d^	9.28 ± 0.175 ^b^	0.000	0.046	0.000
C17:0, mg/g	0.31 ± 0.018 ^c^	0.35 ± 0.005 ^b^	0.40 ± 0.011 ^a^	0.38 ± 0.015 ^ab^	0.39 ± 0.016 ^a^	0.38 ± 0.014 ^a^	0.33 ± 0.006 ^c^	0.001	0.149	0.001
C18:0, mg/g	25.89 ± 0.325 ^e^	27.72 ± 0.306 ^a^	26.53 ± 0.122 ^d^	26.89 ± 0.373 ^cd^	27.18 ± 0.212 ^abc^	27.49 ± 0.255 ^ab^	27.15 ± 0.381 ^bc^	0.034	0.125	0.002
C18:1n9 c, mg/g	111.19 ± 1.391 ^c^	102.71 ± 1.263 ^e^	114.44 ± 0.588 ^b^	109.69 ± 1.573 ^cd^	98.73 ± 0.879 ^f^	118.80 ± 0.860 ^a^	108.37 ± 1.272 ^d^	0.000	0.575	0.000
C18:2n6 c, mg/g	28.78 ± 0.363 ^f^	36.68 ± 0.477 ^b^	31.16 ± 0.100 ^e^	34.86 ± 0.456 ^c^	43.55 ± 0.425 ^a^	28.07 ± 0.252 ^g^	33.29 ± 0.408 ^d^	0.000	0.001	0.000
C18:3n6, mg/g	0.20 ± 0.005 ^bc^	0.22 ± 0.013 ^b^	0.20 ± 0.002 ^bc^	0.20 ± 0.013 ^cd^	0.29 ± 0.023 ^a^	0.17 ± 0.005 ^e^	0.18 ± 0.010 ^de^	0.000	0.221	0.000
C18:3n3, mg/g	0.65 ± 0.014 ^f^	1.06 ± 0.027 ^b^	0.68 ± 0.008 ^e^	1.02 ± 0.011 ^c^	1.54 ± 0.012 ^a^	0.57 ± 0.011 ^g^	0.91 ± 0.009 ^d^	0.000	0.000	0.000
C20:1, mg/g	0.68 ± 0.015 ^cd^	0.66 ± 0.012 ^de^	0.78 ± 0.010 ^b^	0.69 ± 0.012 ^c^	0.63 ± 0.007 ^f^	0.81 ± 0.011 ^a^	0.65 ± 0.005 ^e^	0.000	0.018	0.000
C20:2, mg/g	0.35 ± 0.006 ^f^	0.46 ± 0.017 ^b^	0.41 ± 0.003 ^d^	0.43 ± 0.008 ^c^	0.53 ± 0.012 ^a^	0.38 ± 0.007 ^e^	0.38 ± 0.017 ^e^	0.000	0.840	0.000
C22:0, mg/g	0.22 ± 0.011 ^ab^	0.19 ± 0.020 ^c^	0.24 ± 0.008 ^a^	0.22 ± 0.003 ^ab^	0.21 ± 0.011 ^bc^	0.22 ± 0.007 ^ab^	0.19 ± 0.021 ^c^	0.002	0.102	0.005
C20:3n6, mg/g	0.44 ± 0.019 ^b^	0.50 ± 0.031 ^a^	0.43 ± 0.012 ^b^	0.44 ± 0.005 ^b^	0.49 ± 0.002 ^a^	0.42 ± 0.017 ^b^	0.39 ± 0.019 ^c^	0.000	0.016	0.145
C20:4n6, mg/g	5.18 ± 0.140	5.14 ± 0.08	5.30 ± 0.096	5.29 ± 0.082	5.20 ± 0.109	5.24 ± 0.054	5.21 ± 0.064	0.167	0.539	0.420
C24:1, mg/g	0.12 ± 0.014 ^ab^	0.09 ± 0.014 ^c^	0.14 ± 0.009 ^a^	0.10 ± 0.022 ^bc^	0.09 ± 0.010 ^c^	0.15 ± 0.005 ^a^	0.07 ± 0.023 ^c^	0.000	0.399	0.154
SFA, mg/g	100.26 ± 1.212 ^a^	96.22 ± 1.175 ^b^	99.38 ± 0.526 ^a^	99.01 ± 1.397 ^a^	95.47 ± 0.805 ^b^	99.51 ± 0.716 ^a^	100.66 ± 1.275 ^a^	0.128	0.100	0.086
UFA, mg/g	158.71 ± 2.048 ^bc^	154.91 ± 1.937 ^d^	162.60 ± 0.818 ^a^	161.21 ± 2.268 ^ab^	157.85 ± 1.353 ^cd^	161.21 ± 1.280 ^a^	158.93 ± 1.935 ^bc^	0.002	0.021	0.015
TFA, mg/g	258.97 ± 3.259 ^a^	251.13 ± 3.112 ^b^	261.99 ± 1.344 ^a^	260.22 ± 3.665 ^a^	253.32 ± 2.142 ^b^	260.59 ± 1.995 ^a^	259.59 ± 3.207 ^a^	0.088	0.104	0.067

^1^ Each value is presented as mean ± SD, different superscript (a–d) indicate significant differences (*p* < 0.05). ^2^ C14:0 = myristic acid, C14:1 = myristic oleic acid, C15:0 = pentadecanoic acid, C16:0 = palmitic acid, C16:1 = palmitoleic acid, C17:0 = margaric acid, C18:0 = stearic acid, C18:1n9c = oleic acid, C18:2n6c = linoleic acid, C18:3n6 = methyl linoleate, C18:3n3 = α-linolenic acid methylester, C20:1 = eicosanoic acid, C20:2 = eicosadienoic acid, C22:0 = behenic acid, C20:3n6 = eicosatrienoic acid, C20:4n6 = methyl arachidonate, C24:1 = neuronic acid, SFA = saturated fatty acid, UFA = unsaturated fatty acid, and TFA = total fatty acid. ^3^ Soybean oil 1.5% or 3%: addition of 1.5% or 3% of soybean oil to the base diet, respectively. Lard 1.5% or 3%: addition of 1.5% or 3% of lard to the base diet, respectively. Mixed oils: 1.5% or 3%: addition of 1.5% or 3% of mixed oils to the base diet, respectively.

**Table 6:** Effects of different concentrations of soybean oil, lard, and mixed oils on protein, amino acids, moisture, cholesterol, choline, and phospholipids in raw egg yolk ^1^.

Traits ^2^	Control Group	Concentration ^3^						*p*-Value from ANOVA Mixed Design		
		1.5%			3%					
		Soybean Oil	Lard	Mixed Oils	Soybean Oil	Lard	Mixed Oils	Type	Concentration	Type × Concentration
Asp, mg/g	12.53 ± 0.115 ^b^	12.53 ± 0.115 ^b^	12.17 ± 0.115 ^d^	12.87 ± 0.115 ^a^	12.27 ± 0.115 ^cd^	12.47 ± 0.115 ^bc^	12.37 ± 0.153 ^bcd^	0.002	0.017	0.000
Thr, mg/g	6.70 ± 0.100 ^b^	6.87 ± 0.058 ^ab^	6.67 ± 0.208 ^b^	7.03 ± 0.153 ^a^	6.67 ± 0.058 ^b^	6.87 ± 0.058 ^ab^	6.73 ± 0.153 ^b^	0.213	0.113	0.009
Ser, mg/g	10.47 ± 0.115 ^ab^	10.47 ± 0.058 ^ab^	10.13 ± 0.153 ^c^	10.67 ± 0.115 ^a^	10.20 ± 0.100 ^c^	10.33 ± 0.058 ^bc^	10.17 ± 0.153 ^c^	0.044	0.003	0.000
Glu, mg/g	15.63 ± 0.058 ^ab^	15.40 ± 0.265 ^bc^	15.20 ± 0.200 ^c^	15.73 ± 0.159 ^a^	15.33 ± 0.058 ^bc^	15.53 ± 0.252 ^abc^	15.30 ± 0.100 ^bc^	0.262	0.511	0.007
Gly, mg/g	3.93 ± 0.058 ^bcd^	4.10 ± 0.001 ^a^	3.90 ± 0.100 ^cd^	4.07 ± 0.058 ^ab^	3.83 ± 0.115 ^d^	4.03 ± 0.058 ^abc^	3.97 ± 0.058 ^abcd^	0.409	0.039	0.001
Ala, mg/g	6.70 ± 0.001 ^ab^	6.67 ± 0.115 ^ab^	6.43 ± 0.115 ^c^	6.80 ± 0.001 ^a^	6.43 ± 0.058 ^c^	6.70 ± 0.173 ^ab^	6.60 ± 0.100 ^bc^	0.041	0.258	0.001
Cys, mg/g	1.23 ± 0.058 ^bcd^	1.20 ± 0.100 ^cd^	1.10 ± 0.001 ^d^	1.40 ± 0.173 ^ab^	1.30 ± 0.100 ^abc^	1.33 ± 0.115 ^abc^	1.47 ± 0.058 ^a^	0.004	0.013	0.340
Val, mg/g	7.90 ± 0.173 ^a^	8.03 ± 0.208 ^a^	7.57 ± 0.058 ^b^	8.07 ± 0.115 ^a^	7.80 ± 0.100 ^ab^	7.90 ± 0.265 ^a^	7.87 ± 0.153 ^ab^	0.068	0.677	0.017
Met, mg/g	1.87 ± 0.058 ^a^	1.50 ± 0.001 ^c^	0.62 ± 0.015 ^f^	1.20 ± 0.100 ^e^	1.10 ± 0.100 ^e^	1.33 ± 0.058 ^d^	1.63 ± 0.058 ^b^	0.000	0.000	0.000
IIe, mg/g	6.60 ± 0.100 ^a^	6.53 ± 0.058 ^a^	6.20 ± 0.100 ^b^	6.50 ± 0.173 ^a^	6.47 ± 0.208 ^a^	6.50 ± 0.100 ^a^	6.23 ± 0.058 ^b^	0.111	0.854	0.005
Leu, mg/g	11.27 ± 0.153 ^abc^	11.47 ± 0.115 ^ab^	10.97 ± 0.252 ^c^	11.60 ± 0.200 ^a^	11.20 ± 0.300 ^bc^	11.33 ± 0.153 ^abc^	11.07 ± 0.153 ^c^	0.218	0.146	0.005
Tyr, mg/g	4.63 ± 0.153 ^abc^	4.70 ± 0.001 ^ab^	4.50 ± 0.200 ^bcd^	4.67 ± 0.115 ^abc^	4.37 ± 0.058 ^d^	4.73 ± 0.058 ^a^	4.47 ± 0.058 ^cd^	0.447	0.077	0.002
Phe, mg/g	5.77 ± 0.115	5.77 ± 0.058	5.60 ± 0.300	5.80 ± 0.100	5.60 ± 0.100	5.70 ± 0.100	5.50 ± 0.173	0.911	0.115	0.109
Lys, mg/g	10.27 ± 0.252 ^ab^	10.07 ± 0.153 ^bc^	9.63 ± 0.058 ^d^	10.37 ± 0.153 ^a^	9.87 ± 0.058 ^c^	10.00 ± 0.001 ^c^	9.93 ± 0.058 ^c^	0.002	0.172	0.000
His, mg/g	2.80 ± 0.100 ^d^	3.20 ± 0.100 ^ab^	2.73 ± 0.208 ^d^	3.37 ± 0.115 ^a^	2.97 ± 0.153 ^bcd^	3.13 ± 0.115 ^abc^	2.90 ± 0.100 ^cd^	0.052	0.132	0.000
Arg, mg/g	8.57 ± 0.153 ^bc^	8.87 ± 0.208 ^ab^	8.43 ± 0.208 ^c^	9.00 ± 0.100 ^a^	8.53 ± 0.058 ^bc^	8.63 ± 0.379 ^abc^	8.53 ± 0.153 ^bc^	0.16	0.056	0.031
Pro, mg/g	4.87 ± 0.058 ^ab^	4.90 ± 0.200 ^a^	4.57 ± 0.208 ^bcd^	4.50 ± 0.100 ^cd^	4.33 ± 0.305 ^d^	4.60 ± 0.100 ^abcd^	4.67 ± 0.058 ^abc^	0.927	0.150	0.005
Essential amino acids	50.37 ± 0.379 ^a^	50.23 ± 0.551 ^a^	47.26 ± 0.845 ^c^	50.57 ± 0.635 ^a^	48.70 ± 0.656 ^b^	49.63 ± 0.493 ^ab^	48.97 ± 0.231 ^b^	0.23	0.150	0.080
Non-essential amino acids	71.37 ± 0.252 ^bc^	72.03 ± 0.961 ^ab^	69.17 ± 0.305 ^d^	73.07 ± 0.473 ^a^	69.57 ± 0.723 ^d^	71.50 ± 1.311 ^bc^	70.43 ± 0.651 ^cd^	0.003	0.010	0.084
Protein, mg/g	153.33 ± 3.215a	145.67 ± 3.055 ^cd^	146.67 ± 2.082 ^bcd^	152.00 ± 4.359 ^ab^	144.33 ± 2.082 ^d^	147.67 ± 3.215 ^bcd^	150.33 ± 2.309 ^abc^	0.01	0.645	0.709
Moisture, mg/g	513.00 ± 2.646c	518.00 ± 1.000bc	523.67 ± 6.658ab	501.67 ± 2.082 ^d^	519.67 ± 0.578 ^bc^	532.67 ± 12.220 ^a^	527.33 ± 1.527 ^ab^	0.964	0.073	0.266
Cholesterol, mg/kg	9324.85 ± 70.15 ^c^	11235.58 ± 172.75 ^a^	9276.28 ± 145.59 ^c^	10451.50 ± 121.79 ^b^	10137.68 ± 176.82 ^b^	10195.01 ± 108.52 ^b^	10316.91 ± 195.99 ^b^	0.000	0.532	0.000
Choline, mg/g	7.03 ± 0.115 ^c^	9.98 ± 0.066 ^a^	6.82 ± 0.053 ^d^	5.81 ± 0.096 ^f^	8.55 ± 0.126 ^b^	6.09 ± 0.076 ^e^	6.17 ± 0.148 ^e^	0.000	0.000	0.000
Phospholipids, U/g	11858.40 ± 303.32 ^d^	14738.89 ± 416.27 ^c^	14491.99 ± 303.32 ^c^	16330.02 ± 47.52 ^a^	15589.32 ± 332.61 ^b^	14505.71 ± 365.75 ^c^	16549.48 ± 345.10 ^a^	0.000	0.032	0.098

^1^ Each value is presented as mean ± SD, different superscript (a–f) indicates significant differences (*p* < 0.05). ^2^ Asp = Aspartic acid, Thr = Threonine, Ser = Serine, Glu = Glutamic acid, Gly = Glycine, Ala = Alanine, Cys = Cystine, Val = Valine, Met = Methionine, Ile = Isoleucine, Leu = Leucine, Tyr = Tyrosine, Phe = Phenylalanine, Lys = Lysine, His = Histidine, Val = Valine, Arg = Arginine, and Pro = Proline. ^3^ Soybean oil 1.5% or 3%: addition of 1.5% or 3% of soybean oil to the base diet, respectively. Lard 1.5% or 3%: addition of 1.5% or 3% of lard to the base diet, respectively. Mixed oils: 1.5% or 3%: addition of 1.5% or 3% of mixed oils to the base diet, respectively.

**Table 7:** Effects of different concentrations of soybean oil, lard, and mixed oils on amino acids, protein, and moisture of albumen ^1^.

Traits ^2^	Control Group	Concentration ^3^						*P*-Value from ANOVA Mixed Design		
		1.5%			3%					
		Soybean Oil	Lard	Mixed Oils	Soybean Oil	Lard	Mixed Oils	Type	Concentration	Type × Concentration
Asp, mg/g	8.87 ± 0.058 ^d^	9.73 ± 0.058 ^c^	10.03 ± 0.058 ^b^	9.67 ± 0.058 ^c^	9.80 ± 0.100 ^c^	10.50 ± 0.100 ^a^	10.00 ± 0.100 ^b^	0.000	0.000	0.002
Thr, mg/g	3.93 ± 0.058 ^e^	4.30 ± 0.001 ^cd^	4.43 ± 0.058 ^b^	4.23 ± 0.058 ^d^	4.27 ± 0.058 ^d^	4.63 ± 0.058 ^a^	4.37 ± 0.058 ^bc^	0.000	0.001	0.006
Ser, mg/g	5.63 ± 0.058 ^e^	6.27 ± 0.058 ^bc^	6.33 ± 0.058 ^b^	6.07 ± 0.058 ^d^	6.17 ± 0.058 ^cd^	6.77 ± 0.058 ^a^	6.27 ± 0.058 ^bc^	0.000	0.000	0.000
Glu, mg/g	11.50 ± 0.001 ^e^	12.67 ± 0.153 ^c^	12.97 ± 0.058 ^b^	12.37 ± 0.115 ^d^	12.67 ± 0.252 ^c^	13.67 ± 0.115 ^a^	12.87 ± 0.115 ^bc^	0.000	0.000	0.002
Gly, mg/g	2.97 ± 0.058 ^d^	3.30 ± 0.001 ^c^	3.40 ± 0.001 ^b^	3.30 ± 0.001 ^c^	3.37 ± 0.058 ^b^	3.50 ± 0.001 ^a^	3.40 ± 0.001 ^b^	0.000	0.000	0.571
Ala, mg/g	5.13 ± 0.115 ^c^	5.70 ± 0.100 ^b^	5.80 ± 0.100 ^b^	5.60 ± 0.100 ^b^	5.77 ± 0.153 ^b^	6.13 ± 0.115 ^a^	5.73 ± 0.153 ^b^	0.002	0.008	0.178
Cys, mg/g	2.13 ± 0.058 ^d^	2.20 ± 0.001 ^cd^	2.57 ± 0.058 ^a^	2.27 ± 0.058 ^c^	2.50 ± 0.001 ^ab^	2.47 ± 0.058 ^ab^	2.40 ± 0.100 ^b^	0.000	0.001	0.000
Val, mg/g	5.80 ± 0.001 ^e^	6.47 ± 0.058 ^c^	6.67 ± 0.058 ^b^	6.27 ± 0.058 ^d^	6.47 ± 0.058 ^c^	6.90 ± 0.100 ^a^	6.60 ± 0.100 ^b^	0.000	0.000	0.003
Met, mg/g	3.23 ± 0.058 ^d^	3.47 ± 0.058 ^c^	3.47 ± 0.058 ^c^	3.47 ± 0.058 ^c^	3.60 ± 0.100 ^b^	3.93 ± 0.058 ^a^	3.60 ± 0.001 ^b^	0.000	0.000	0.000
IIe, mg/g	4.37 ± 0.115 ^e^	4.73 ± 0.058 ^cd^	5.03 ± 0.058 ^a^	4.63 ± 0.058 ^d^	4.83 ± 0.058 ^bc^	5.10 ± 0.001 ^a^	4.87 ± 0.058 ^b^	0.000	0.001	0.100
Leu, mg/g	7.23 ± 0.058 ^f^	7.97 ± 0.058 ^d^	8.23 ± 0.058 ^b^	7.83 ± 0.058 ^e^	8.00 ± 0.001 ^cd^	8.60 ± 0.001 ^a^	8.07 ± 0.058 ^c^	0.000	0.000	0.000
Tyr, mg/g	2.97 ± 0.115 ^c^	3.07 ± 0.058 ^c^	3.47 ± 0.058 ^a^	3.03 ± 0.058 ^c^	3.23 ± 0.058 ^b^	3.40 ± 0.001 ^a^	3.23 ± 0.058 ^b^	0.000	0.006	0.006
Phe, mg/g	5.17 ± 0.115 ^d^	5.80 ± 0.001 ^bc^	5.90 ± 0.100 ^b^	5.70 ± 0.001 ^c^	5.90 ± 0.001 ^b^	6.20 ± 0.001 ^a^	5.77 ± 0.058 ^c^	0.000	0.000	0.011
Lys, mg/g	5.90 ± 0.100 ^d^	6.57 ± 0.058 ^c^	6.70 ± 0.100 ^b^	6.43 ± 0.058 ^c^	6.53 ± 0.058 ^c^	7.03 ± 0.058 ^a^	6.57 ± 0.058 ^c^	0.000	0.001	0.002
His, mg/g	1.83 ± 0.058 ^c^	2.10 ± 0.100 ^ab^	2.13 ± 0.058 ^ab^	2.03 ± 0.115 ^b^	2.10 ± 0.100 ^ab^	2.20 ± 0.100 ^a^	2.07 ± 0.058 ^ab^	0.066	0.384	0.768
Arg, mg/g	4.67 ± 0.115 ^e^	5.03 ± 0.153 ^cd^	5.27 ± 0.115 ^b^	5.00 ± 0.001 ^d^	5.07 ± 0.058 ^cd^	5.50 ± 0.100 ^a^	5.20 ± 0.100 ^bc^	0.000	0.006	0.228
Pro, mg/g	3.03 ± 0.115 ^d^	3.27 ± 0.058 ^c^	3.33 ± 0.058 ^bc^	3.27 ± 0.058 ^c^	3.30 ± 0.100 ^bc^	3.63 ± 0.058 ^a^	3.43 ± 0.058 ^b^	0.001	0.000	0.028
Essential amino acids	35.63 ± 0.493 ^f^	39.30 ± 0.173 ^d^	40.43 ± 0.058 ^b^	38.57 ± 0.115 ^e^	39.60 ± 0.300cd	42.40 ± 0.100 ^a^	39.83 ± 0.305 ^c^	0.002	0.000	0.010
Non-essential amino acids	48.73 ± 0.153 ^g^	53.33 ± 0.231 ^e^	55.30 ± 0.173 ^b^	52.60 ± 0.346 ^f^	53.97 ± 0.551d	57.77 ± 0.289 ^a^	54.60 ± 0.436 ^c^	0.005	0.005	0.000
Protein, mg/g	101.70 ± 3.157 ^b^	105.00 ± 3.606 ^ab^	105.67 ± 2.517 ^ab^	105.33 ± 2.309 ^ab^	106.33 ± 0.577ab	107.67 ± 2.082 ^a^	103.67 ± 2.517 ^ab^	0.366	0.651	0.437
Moisture, mg/g	877.00 ± 4.000 ^b^	877.00 ± 1.000 ^b^	868.00 ± 1.732 ^c^	877.33 ± 2.517 ^b^	883.33 ± 0.577a	869.67 ± 1.527 ^c^	882.00 ± 1.732 ^a^	0.000	0.001	0.196

^1^ Each value is presented as mean ± SD, different superscript (a–f) indicates significant differences (*p* < 0.05). ^2^ Asp = Aspartic acid, Thr = Threonine, Ser = Serine, Glu = Glutamic acid, Gly = Glycine, Ala = Alanine, Cys = Cystine, Val = Valine, Met = Methionine, Ile = Isoleucine, Leu = Leucine, Tyr = Tyrosine, Phe = Phenylalanine, Lys = Lysine, His = Histidine, Val = Valine, Arg = Arginine, and Pro = Proline. ^3^ Soybean oil 1.5% or 3%: addition of 1.5% or 3% of soybean oil to the base diet, respectively. Lard 1.5% or 3%: addition of 1.5% or 3% of lard to the base diet, respectively. Mixed oils: 1.5% or 3%: addition of 1.5% or 3% of mixed oils to the base diet, respectively.

**Table 8:** Effect of different concentrations of soybean oil, lard, and mixed oils on the texture profile analysis of cooked egg yolk ^1^.

Traits	Control Group	Concentration ^2^						*p*-Value from ANOVA Mixed Design		
		1.5%			3%					
		Soybean Oil	Lard	Mixed Oils	Soybean Oil	Lard	Mixed Oils	Type	Concentration	Type × Concentration
Hardness, g	142.86 ± 22.847 ^d^	158.75 ± 19.975 ^cd^	170.69 ± 18.584 ^ab^	196.46 ± 22.560 ^a^	189.36 ± 28.124 ^a^	197.24 ± 20.473 ^a^	182.66 ± 18.380 ^bc^	0.260	0.203	0.000
Springiness, mm	0.90 ± 0.017 ^a^	0.88 ± 0.027 ^ab^	0.87 ± 0.034 ^ab^	0.90 ± 0.034 ^a^	0.85 ± 0.075 ^b^	0.89 ± 0.017 ^ab^	0.89 ± 0.022 ^ab^	0.081	0.599	0.087
Cohesiveness	0.90 ± 0.024 ^ab^	0.89 ± 0.042 ^b^	0.87 ± 0.044 ^b^	0.92 ± 0.057 ^a^	0.87 ± 0.036 ^b^	0.88 ± 0.028 ^b^	0.89 ± 0.017 ^b^	0.038	0.218	0.126
Gumminess	127.91 ± 20.246 ^e^	140.83 ± 18.997 ^de^	159.03 ± 19.768 ^bcd^	180.42 ± 26.257 ^a^	166.01 ± 30.330 ^abc^	174.48 ± 20.589 ^ab^	151.61 ± 16.269 ^cd^	0.037	0.437	0.000
Chewiness	115.47 ± 18.607 ^e^	124.78 ± 17.999 ^de^	138.59 ± 20.225 ^bcd^	162.18 ± 28.289 ^a^	145.03 ± 29.867 ^abc^	155.44 ± 20.819 ^ab^	134.84 ± 16.337 ^cde^	0.037	0.518	0.000
Resilience	0.63 ± 0.025 ^a^	0.61 ± 0.025 ^b^	0.58 ± 0.041 ^b^	0.60 ± 0.034 ^b^	0.58 ± 0.038 ^b^	0.60 ± 0.018 ^b^	0.61 ± 0.024 ^b^	0.406	0.935	0.007

^1^ Each value is presented as mean ± SD, different superscript (a–e) indicates significant differences (*p* < 0.05). ^2^ Soybean oil 1.5% or 3%: addition of 1.5% or 3% of soybean oil to the base diet, respectively. Lard 1.5% or 3%: addition of 1.5% or 3% of lard to the base diet, respectively. Mixed oils: 1.5% or 3%: addition of 1.5% or 3% of mixed oils to the base diet, respectively.

## Data Availability

The data that support the findings of this study are available on the request from the corresponding author. The data are not publicly available due to privacy or ethical restrictions.
